# Measuring transformative virtual reality experiences in children’s drawings

**DOI:** 10.3758/s13421-024-01575-y

**Published:** 2024-05-08

**Authors:** H. Anna T. van Limpt-Broers, Marie Postma, Max M. Louwerse

**Affiliations:** Department of Cognitive Science and Artificial Intelligence, Tilburg University, PO Box 90153, 5000 LE Tilburg, The Netherlands

**Keywords:** Transformative experience, Awe, Drawings, Overview effect, Children

## Abstract

Transformative experiences in an individual’s life have a lasting impact on identity, belief system, and values. At the core of these experiences is the complex emotion of awe that promotes learning, making it worthwhile to study from an educational point of view. Drawing studies may provide a useful measure of awe in children—one that is more intuitive and attractive than questionnaires alone. Previous studies conducted with adults indicated that the diminished self, associated with transformative experiences, manifests in an actual decrease in size for figures representing the self in drawings. In the current study, self-representation was investigated in drawings of 10- to 12-year-old primary school children within the context of an immersive virtual reality (VR) experience that elicits the overview effect, known to lead to an intense apperception of awe. We did not replicate the adult findings regarding self-size in this younger age group. However, details and complexity in children’s drawings appeared to be impacted by the awe-elicitation procedure in VR. These elements subsequently correlated to learning gains instead of the overview effect, indicating that this measure could be linked to cognitive ability. The findings of the current study contribute to a better understanding of how drawings reflect self-transcendental experiences; however, they also reveal that in younger age groups, they are not necessarily reflected in decreased self-size.

Transformative experiences can be characterized as pivotal moments in life that lead to a fundamental change in personal identity, core preferences, desires, self-awareness, and beliefs, as well as the perception of the world (Paul & Quiggin, 2020). Examples are near-death (Martial et al., 2021; Taves, 2020; Taylor, 2018), out-of-body, and psychedelic experiences (Nour et al., 2016), but also encounters with forces of nature and peak life events. A shared dimension of all such events is that they frequently result in feelings of connectedness to others and feeling small (Chirico et al., 2022). Transformative experiences in education are defined as students acquiring knowledge through experiences that change their view of themselves and the world around them (Gaggioli, 2016; Paul & Quiggin, 2020). These experiences often involve critical reflection to make sense of what transpired (Wee, 2019), which can be referred to as a need for accommodation. The need for accommodation is often caused by confusion about a phenomenon, which results in failure of aligning current mental structures to a new experience. This can subsequently lead to a drive for expanding knowledge and understanding (Keltner & Haidt, 2003). This critical reflection may be divided in (a) motivated use whereby knowledge is applied in context, (b) an expansion of perception through which the world is understood differently, and (c) experiential value whereby knowledge is valued in other contexts and is recognized as relevant to personal identities (Littrell et al., 2022; Pugh et al., 2010). If it involves a disruptive experience, it is called an epiphany (Yacek & Gary, 2020).

The emotion that appears to lie at the core of transformative experiences is awe. This emotion mixes wonder and fear with an overwhelming feeling of admiration (Krenzer et al., 2020). Situations that comprise an element of *vastness* may lead to awe. Vastness refers to either physical size, such as a beautiful view from a mountain like Yosemite National Park (Bai, et al., 2017), or to conceptual size, such as fame, authority, prestige, or inspiring behavior by a loved one (Graziosi & Yaden, 2021). Awe experiences have also been reported for both events of high personal importance such as the birth of a child, and cognitively complex phenomena which are hard to comprehend from the point of view of an individual’s belief system, as may occur in an educational context (Keltner & Haidt, 2003; Valdesolo et al., 2017; Weger & Wagemann, 2021). Similar to what has been identified for transformative experiences (Mezirow, 1997, 2000), awe-inspiring events lead to a need for accommodation by challenging current mental schemas to understand the world and the self in a novel way (Anderson et al., 2020; Cuzzolino, 2019; McPhetres, 2019; Valdesolo et al., 2017; van Limpt-Broers et al., 2020b). Scientists describe the emotion of awe as a continuous motivator in their work (Cuzzolino, 2019), as being awe-inclined leads to curiosity and does not limit learning to a specific topic (Anderson et al., 2020).

Given that transformative experiences are typically rare and spontaneous, attempts have been made to induce events that trigger awe in controlled settings. Past research demonstrated that computer-generated stimuli such as virtual reality (VR) simulations can spark awe (Chirico et al., 2017; Gallagher et al., 2014; McPhetres & Shtulman, 2021; Quesnel & Riecke, 2017; Stepanova et al., 2018). For instance, van Limpt-Broers et al. (2020a, b) found evidence of the emotion being elicited using a 360° view of planet Earth from space in VR. In a VR study by Nelson-Coffey et al. (2019), a view of Earth from space resulted in feelings of gratitude, love, and optimism in participants, alongside the emotion of awe.

## Overview effect

Observing Earth from space is a transformative experience that leads to a profound feeling of awe, wonder, and self-transcendence, reported by many astronauts, and is called “the overview effect” (White, 2014). It can trigger a shift in perspective towards greater interconnectedness of Earth’s inhabitants, and environmental awareness. The effect is long lasting, and more powerful than ‘regular’ instances of awe (Yaden et al., 2016). The overview effect is considered to be the most prototypical case of awe; therefore, it is always accompanied by a profound apperception of this emotion (Chirico, Ferrise et al., 2018a; Stepanova et al., 2019; Yaden, et al., 2016). The psychological mechanisms behind the overview effect, also studied using simulated astronaut experiences in VR (Gallagher et al., 2014; van Limpt-Broers et al., 2020a; Yaden et al., 2016), suggest that individual differences such as a need for cognition and religiousness may play a role in the experience (Gallagher et al., 2014).

### Small-self

An important element of the overview effect, awe, and other self-transcendental experiences is the reported feeling that all living beings on our fragile planet are connected to each other. This interconnectedness arises when the rigid boundaries of the ego dissolve, allowing for the self and others to unify (Jennings et al., 2005). The “self” refers to a range of mental phenomena that are present in every normal waking human, including self-awareness, the use of possessive pronouns such as “I” and “me,” a sense of ownership over the self, and theory of mind (Lebedev et al., 2015). When the focus on the self diminishes, the perception of greatness outside the self is emphasized (Shiota et al., 2007).^1^ Feeling small correlates with self-esteem, power, status, self-efficacy and self-entitlement (Bai et al., 2017), and may represent a development of personality (Perlin & Li, 2020). It also leads to a feeling of oneness with others, and the environment, describing a “unitive experience” (Nour et al., 2016; Shiota et al., 2007; Martial et al., 2021). A counterforce to “connectedness” is the ego, which is why ego-dissolution leads to oneness (Carhart-Harris et al., 2018).

Interconnectedness was found to increase pro-sociality and generosity, moving away from personal interest and towards a concern for the welfare of other people and the community (Bai et al., 2017; Piff et al., 2015; Martial et al., 2021; Yang et al., 2018). Pro-sociality generally entails doing something that is beneficial to others, such as donating time or money, helping, sharing, cooperating, and volunteering (Guan et al., 2019). In one experiment, participants felt a reduced sense of self after watching awe videos, which yielded a more significant donation of money compared with a control condition (Guan et al., 2019). The oneness with the world also increases the sense of belonging, or connectedness to nature, positively influencing ecological behavior and improving personal physical and psychological well-being (Yang et al., 2018).

### Measuring awe and small-self

For experimental measures of awe, there are at least four perspectives that need to be considered: (1) predisposition, (2) immediate self-reports, (3) physiological measures, and (4) pictorial measures. Firstly, predisposition focuses on the natural tendency for someone to experience awe. Someone with a high disposition will more quickly feel awed in situations that meet the conditions to elicit this complex emotion. It has been measured with questionnaires such as the Dispositional Positive Emotion Scale (DPES) questionnaire (Shiota et al., 2006) and the Trait Respect-Related Emotions Scale (TRESS) questionnaire (Nakayama et al., 2020). The second perspective concerns the “right now” or situational awe, typically measured with a single item for the emotion (Piff et al., 2015; Rudd et al., 2012), or with a more elaborate questionnaire that covers the subcomponents of awe, such as perceived vastness, physical sensations, the small-self and the unitive experience (Krenzer et al., 2020; Yaden et al., 2019). These subcomponents indicate how complex this emotion is. Awe is usually considered on a scale, as one individual can feel slightly awed by a stimulus that elicits a great amount of awe in a different individual. The subcomponents have also been measured separately, using, for example, the Ego-Dissolution Inventory (Nour et al., 2016). The third aspect concerns physiological indicators of awe that can be assessed by measuring goosebumps with a camera (Maruskin et al., 2012; McPhetres & Shtulman, 2021), heart-rate variability, respiration rate, and skin conductance with sensors (Chirico et al., 2017; Shiota et al., 2011), or measures of brain activity on the scalp (Chirico et al., 2020; Hu et al., 2017; Reinerman-Jones et al., 2013; Takano & Nomura, 2022) as well as neural activity for ego-dissolution (Lebedev et al., 2015). Finally, a fourth perspective on experimental measures of awe concerns assessments of the small-self within pictorial methods that make use of representations of the human body (Van Elk et al., 2016), a range of circles (Colantonio & Bonawitz, 2018; Sawada & Nomura, 2020), an open field where participants could draw themselves (Bai et al., 2017), or size within photograph selfies (Sturm et al., 2020).

### Measuring awe in children

For young participants—for instance, 10–12 year olds—trait-level and situational awe questionnaires for adults, albeit commonly used in research, pose difficulties. One common problem is the language of questionnaire items, where children may not immediately comprehend the underlying meaning because of task complexity or vocabulary less appropriate for their age (Pouscoulous & Tomasello, 2019). Depending on the child’s level of comprehension, they may also have difficulty understanding “vague” words and negatively stated questions. While some children from a young age may already comprehend nonliteral language (Falkum, 2022; Pouscoulous & Tomasello, 2019), it is known to be difficult for second language learners (Hoang, 2014; Nacey, 2017; Zhou et al., 2022). A difference in background knowledge of the second language can play a role in language comprehension as well (Burgoyne et al., 2013). Moreover, data quality can also be effected when questionnaires are too long (Bell, 2007; Borgers et al., 2000; de Leeuw et al., 2004; Mellor & Moore, 2014). These difficulties do not invalidate the use of questionnaires in children but do make an investigation into (complementary) alternatives desirable.

Pictorial measures may enrich information from questionnaires as they are language independent. Drawing is commonly used to find out how children interpret the world. It can be considered a “child centered” method because it is an enjoyable, familiar activity (Johnson et al., 2012), and a natural way for children to convey their innermost thoughts, desires, and emotions (Farokhi & Hashemi, 2011; Hsu, 2014). Even participants who are unable to use words to describe their feelings or experiences can usually convey their thoughts on paper through drawings (Hamama & Ronen, 2009). Children are already able to communicate emotions through drawing from the age of 4 or 5 (Bonoti & Misailidi, 2006). Between the ages of 9 and 11, they strive to draw using more details, actions, and accurate representations of the world, humans and the self, using space and perspective (Walker, 2007). Drawing studies therefore provide a good way to enrich and confirm information from questionnaires, especially for young participants, and may eventually serve as a substitute when analysis guidelines are more established.

Despite the potential benefits of drawings as a measure for awe, interpreting human figure representations, especially of those created by children, is far from straightforward. Analyses have often focused on the size of the drawings. For instance, larger human figure drawings have been reported to depict higher importance (Walker, 2012). A child’s self-confidence may also be reflected in figure size, along with the position of the figure on paper and the strength of the used utensils (Hamama & Ronen, 2009). Aside from self-confidence, larger size can also be a reflection of positive perception, where a friendly person would be drawn larger than a nasty one (Burkitt et al., 2009). At the same time, however, figures that are drawn larger and more exaggerated can in some contexts also be interpreted as more aggressive or overactive. Children drawing smaller figures may feel a lack of competence, shame, fear, and depression (Farokhi & Hashemi, 2011). To make matters worse, the size of a drawn self-figure can be partially determined by cultural background, where group identification with a greater sense of belonging leads to a more stable self and thus a larger figure in Asian cultures compared with North American cultures (La Voy et al., 2001; Yap et al., 2022).

Bai et al. (2017) presents the most comprehensive research of awe in drawings. They found strong evidence for daily, in vivo, and lab experiences of awe diminishing the sense of self in a between-participants design reflected in smaller figures. Their results indicated that attention was shifted away from individual interests and concerns. The Bai et al. research stands out in the number of studies (6), and participants (2,137), the combination between in vivo and lab experiences, as well as the cross-cultural investigation (USA and China), and the age ranges (18–92 years of age). However, in those cases where Bai et al. used stimuli in their experiments, 5-min montage of clips of natural phenomena were used. Whether more profound immersive experiences—the overview effect in a 360 degrees 3D virtual reality simulation—yields awe the same way or more profoundly is yet unclear. Moreover, it is uncertain whether the finding of a diminished sense of self is visible from pre- to post-awe exposure. Finally, particularly for children drawing might be a good substitute for questionnaires when measuring awe. However, we do not know to what extent drawings produce similar results in children as they do in adults. To answer these questions, the current research explored changes in self-perception related to a VR-induced overview effect experience in children, using both traditional questionnaire techniques as well as drawings in a within-participants study design.

## Current study

In the current study, the overview effect and awe were induced through a virtual reality journey to space in a population of 10- to 12-year-old children and assessed using both questionnaires and drawings. This research was embedded in an educational program created by the nonprofit organization SpaceBuzz. This program consisted of a preflight “astronaut training,” composed of multiple playful educational activities in the classroom, followed by a VR journey through space in a replica rocket ship that arrived at the children’s school. Finally, postflight lessons in school allowed children to reflect on the knowledge and experience they gained. In previous research it was established that the SpaceBuzz simulation yielded the overview effect and feelings of awe in children (van Limpt-Broers et al., 2020a), making it a useful experimental environment to investigate whether awe can also be measured using a drawing method. In the current study, we hypothesized that a stronger experience of the overview effect, and subsequently feelings of awe, would result in a size reduction of the self-drawing (Bai et al., 2017). In addition, because awe experiences are assumed to promote learning (van Limpt-Broers et al., 2020b), we predicted a relation between the strength of the overview effect, awe (measured in the drawings), and learning gains.

## Method

### Participants

Participants (*N* = 100) were recruited at the United World College Maastricht, an international school in the Southern part of the Netherlands. One participant was removed from the sample because of an experimental error, resulting in a final sample of 99 (*M*_age_ = 11.24 years, *SD*_age_ = 0.69; *Min*_age_ = 10.00 years; *Max*_age_ = 12.00 years; 47 male, 41 female, and 11 gender not reported). The country of residence for all participants was the Netherlands; however, children’s country of origin and ethnicity was not recorded, to safeguard privacy and anonymity, and, subsequently, cannot be reported. The school hosts about 900 students, with around 100 nationalities. They support children to increase their language skills for three languages—namely, English, which is the main language used at the school, as well as Dutch, the host-country’s language, and giving support for children to keep speaking their native language. The level of English proficiency thus differs per participant. Simpler vocabulary within our research ensures that all children can comprehend the questions. Prior to the experiment, written consent to participate was obtained from the legal guardians. The study was approved by the Ethical Review Board of Tilburg University (REDC # 2019/04a). A post hoc power analysis using G*Power (Faul et al., 2007) indicated 99% power to detect medium-sized effects (*d* = .5) in one-sample *t* tests, with a .05 two-tailed Type I error probability.

### Materials

**Fig. 1:**
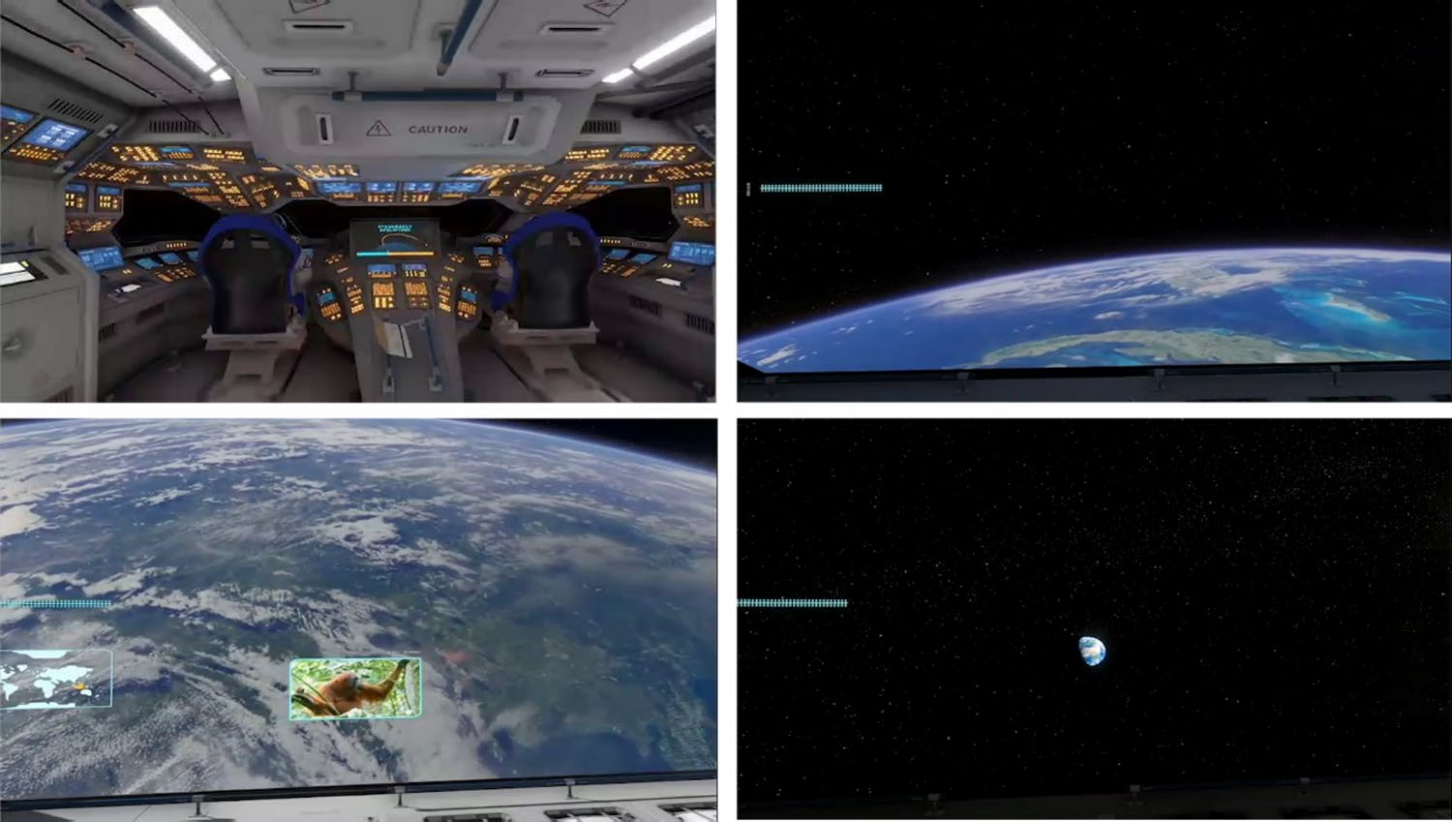
Screenshots of the simulation

The simulation was presented on VR headsets (HTC Vive Pros with resolution: 1,440 × 1,600 pixels per eye, 615 PPI, 3D Spatial Audio, refresh rate of 90 Hz) in the SpaceBuzz rocket replica. Both the size of the rocket ship (13 feet high, 8 feet wide, and 50 feet long) and its design served to make the experience more realistic. The interior contained nine chairs that rotated, vibrated, and tilted synchronized with the events in the simulation. The virtual journey to space was created in Unity and had a total length of 14 minutes and 25 seconds. It was narrated by European Space Agency (ESA) astronaut André Kuipers with prerecorded audio and video. The simulation focused on aspects that are of importance for the overview effect. It started with a launch into orbit around Earth. A vibrating and tilting chair enhanced the experience. Next, the doors of the rocket ship opened, and the young astronauts viewed planet Earth in the vastness of space. Topics such as Earth’s population, natural phenomena, environmental issues, and satellites were discussed. At the end of the simulation, during a short trip to the Moon, an emotionally charged message was played that addressed the interconnectedness of all beings of “Spaceship Earth.” The simulation was concluded by safely returning to Earth. See Fig. 1 for stills of the simulation.^2^

#### Overview effect

The Positive Changes from Space Travel subscale (PCST) of the Positive Effects of Being in Space Questionnaire was used to measure the overview effect (Ihle et al., 2006). The questionnaire consisted of thirteen 5-point scale items, with subcomponents Perceptions of Earth, Perceptions of Space, and Changes in Daily Life. The items were adapted for children in line with recommendations in the literature, showing that easier language is more inclusive of any level of English in the bilingual participant population (Bell, 2007; Borgers et al., 2000; Nacey, 2017; Van Kesteren et al., 2003). To allow for pre- and posttests, two versions of the questionnaires were created consisting of synonyms. Questionnaires were based on background knowledge and language skills for students of this age for the Dutch curriculum (Kuiken & Droge, 2010), considering school location. These were counterbalanced in the pre- and posttests. For instance, an item from the original Positive Effects of Being in Space Questionnaire’s subcomponent Perceptions of Earth, *“I increased my involvement in environmental causes.”* was changed to the following, more relatable, and understandable two versions: *“Do you take good care of the Earth (such as cleaning up garbage or turning off the lights when you leave your room)?”* in one version, and “*Do you take good care of the environment (such as throwing things in the trash or turning off the tap while brushing your teeth)?”* in the other. An item from the original Positive Effects of Being in Space Questionnaire’s subcomponent Changes in Daily Life, *“I increased my involvement in political activities.”* was changed to the following two versions: *“Do you want to know more about what the government does?”* in one version, and “*Do you want to learn about what political parties do?”* in the other. See Appendix Table 4 for the full questionnaire. Note that the meaning of the original question was intact, but paraphrases were used with simpler wording. Cronbach’s alpha was acceptable (Taber, 2018) at .70 and .73 for pre- and postmeasures, respectively, indicating sufficient internal consistency.^3^

#### Awe

Before and after the VR space journey participants were asked to draw a picture of “themselves as an astronaut” in an empty rectangle with grass at the bottom, and either the sun or the moon (counterbalanced for the pre- or postmeasures) at the top (see Appendix Fig. 4). Participants were instructed as follows: “*Draw yourself as an astronaut. Draw yourself in the image below. You can draw anything you want, and the picture can be anything, as long as you are in it.”* The cognitive task was therefore similar to Bai et al. (2017), including only the drawing instructions and field. Following Bai et al. (2017), in addition to the drawing assignment, no questionnaires on awe were specifically asked. Note that the Overview Effect questionnaire served as a proxy for awe already, as it is considered a prototypical case of the emotion (Chirico et al., 2017). Moreover, omitting a longer questionnaire on awe, such as the AWE-S scale (Yaden et al., 2019) avoided a possible priming effect on small-self within drawings with questions, such as *“I felt small compared with everything else,”* and ensured that the total experiment would not exceed an hour.

Predrawings were supervised by the teacher in class, and children used their own drawing materials; most participants used line-art whether they used a (colored) pencil or pen. Postdrawings were supervised by the experimenter. To measure perceived self-size, the height of the character from the longest leg to the top of the head including clothing and hats was recorded in centimeters with one decimal. Because drawings can vary considerably in size between participants the data was normalized using the following equation:

1 $$Drawing\ gains=\frac{\frac{post}{(pre+post)}- \frac{pre}{(pre+post)}}{1-\frac{pre}{(pre+post)}} .$$

#### Learning

Two knowledge tests based on educational content that was covered within the VR simulation about space, appropriate for 10- to 12-year-old children, were created and used as pre- and postmeasures. They consisted of 16 multiple-choice questions each. Both versions were counterbalanced on topics (see Appendix 3). All questionnaires were evaluated by all authors for consistency. The pre- and posttests were used to calculate learning gains as a proxy for learning (Angel-urdinola et al., 2021; Luo et al., 2021), which were calculated with the following equation (Craig et al., 2004; van Limpt-Broers et al., 2020a):

2 $$learning\ Gains=\frac{\left(proportion\ correct\ posttest\right)-(proportion\ correct\ pretest)}{1-(proportion\ correct\ pretest)}.$$

#### Procedure of children’s experiment

Prior to the experiment, participants followed a week’s worth of space related classes as part of the SpaceBuzz educational program, so they all had a minimum knowledge base about space. Differences in prior knowledge were controlled for by administering pre- and postknowledge tests and calculating individual learning gains.

Two school classes from the same school participated in the study. Pre- and posttests were counterbalanced between classes. Participants started by filling out demographic information, the overview effect questionnaire, the drawing assignment for awe, and the knowledge test, under the supervision of their own teacher. Next, participants experienced the VR simulation in the rocket ship in groups of nine. A SpaceBuzz attendant helped with the VR headsets and provided further in-rocket assistance. Finally, participants who finished the experience filled in the remainder of the questionnaires: the overview effect questionnaire, the drawing assignment for awe, and the postknowledge test under supervision of the first author. Questionnaires were administered on paper, resulting in some participants skipping a couple of questions and resulting in missing data.

#### Procedure of drawing ratings

Following up the beforementioned experiment was a rater study, based on preliminary results showing that the drawings of awed participants did not reflect a reduction in self-size alone. Therefore, it was investigated whether children might portray awe in a different way than self-size. Drawings after all show more than only figure height and are a rich source of information. Extracting quality or content-related data from drawings is often done by raters or authors (Cox et al., 2001; Groth-Marnat & Roberts, 1998; Jurovatý et al., 2022). These ratings can then be compared with standardized measures such as questionnaire answers, potentially verifying the use of drawings as an alternative measurement of awe for a young population. It was hypothesized that annotated scores for awe increase from pre- to postdrawings as identified by independent raters.

To rate the drawings collected from the participants, raters who were blind to the design of the experiment (*N* = 120), were recruited online through the Tilburg School of Humanities and Digital Sciences participant pool (*M*_age_ = 21.40 years; *SD*_age_ = 3.37; 94 female, 25 male, one not reported). Separate approval for this part of the study was granted by the Ethical Review Board of Tilburg University (REDC # 2019/04b). Raters consented for participation and received credit upon completing the online questionnaire.

Raters signed up for the experiment on the department’s participant pool platform. They were redirected to the online survey, using Qualtrics (www.qualtrics.com). The survey started with a brief demographic questionnaire. Raters were then instructed to read the explanations of awe from Bai et al. (2017), Gordon et al. (2017), Graziosi & Yaden (2021), and Nakayama et al. (2020), and were asked to reproduce the definition for awe in their own words as a check of their understanding. Upon inspection of their answers all described the feeling of awe correctly, summarizing the definitions they had read earlier.

To investigate whether awe can be detected in individual drawings, raters determined whether the drawing represented awe for each single drawing, referred to as the single-drawing condition. Raters (*N* = 59) in the single-drawing condition judged all 198 drawings from the main study one at a time, presented in random order. They were asked to indicate whether a drawing depicted “awe” or “no awe.” This was to gain insight into what in drawings would depict awe if viewed alone. As awe—like any other emotion—is not a binary state, the annotated scores were summed over raters so that the level of awe per drawing could be estimated.

However, one might argue that ratings on single drawings are problematic; because awe is generally dependent on context, on an individual’s predisposition to awe, and individual differences on drawing style, drawings were also judged by raters side-by-side, referred to as the paired-drawings condition. Raters (*N* = 61) judged 99 paired drawings, each pair from the same child, side-by-side in random order to avoid a left-right effect. This binary task again yielded a scale of assumed awe when answers of all raters were summed. Because there are a large number of individual differences between children and their drawings, this paired rating might be a better reflection of actual awe in drawings, than when judged separately, considering that a “neutral” emotional state can be compared with an “emotional state of awe.”

The trials were preceded by a random selection of five drawings from the full set, for practice purposes. Annotated scores from these ratings were summed, resulting in an annotated awe score for single-drawings (between 0 and 59), and a score for paired drawings (between 0 and 61). These scores were used as a ratio scale, where a higher score indicated the likelihood that awe was represented in a drawing.

## Results

### The small-self in children’s drawings

Because the data was not normally distributed, non-parametric tests were used. A Wilcoxon signed rank test was conducted for each variable to determine whether an effect occurred from pre- to posttest. Correlations are Spearman correlations.

#### Overview effect

Results revealed the baseline for the overview effect to be *Mdn* = 3.92, on a 5-point scale, with 3 as the center point. A Wilcoxon signed-rank test indicated that the post measure for the overview effect was significantly higher than the baseline, (*Mdn* = 4.08)*, T* = 3577, *z* = −5.336, *p* < .001. These results suggest that the participants experienced the overview effect after viewing Earth from space in VR, replicating the findings reported in van Limpt-Broers et al. (2020a, b). Given that the overview effect is the most prototypical case of an awe experience (Chirico, Ferrise et al., 2018a), it can be concluded that awe has been successfully elicited as well.

#### Awe as measured by small-self

To assess awe in drawings using human-figure height, results revealed a decrease in drawing size from pretest (*Mdn* = 5.40) to posttest (*Mdn* = 5.30), which is the expected direction for a larger feeling of awe. Contrary to our predictions, informed by Bai et al. (2017), a Wilcoxon signed-rank test showed this difference did not reach significance, *T* = 2163, *z* = −.626, *p* = .793.

#### Learning

Proportional learning gains scores (*Mdn* = 0.14) were significantly different from 0 using a Wilcoxon signed-ranked test, *T* = 2187, *z* = 2.722, *p* = .006, demonstrating participants gained knowledge from the VR journey, replicating van Limpt-Broers et al. (2020b).

### Rating of children’s drawings

#### Awe as measured by raters

Annotated awe scores given by raters in the single-drawing condition did not show more awe from pretest (*Mdn* = 27) to posttest (*Mdn* = 26), with a Wilcoxon signed-rank test, *T* = 2423, *z* = −721, *p* = .471. Raters in the paired-drawings condition also did not give a significantly higher score from pretest (*Mdn* = 27) to posttest (*Mdn* = 34), with a Wilcoxon signed-rank test, *T* = 2686, *z* = −.737, *p* = .461. For an overview of all results, see Table 1.

**Table 1:** Overview of experimental results, *z* from Wilcoxon signed rank test comparing the gains score to 0

	Pre			Post			Proportional Gains			
	Med	Min	Max	Med	Min	Max	Med	Min	Max	*Z*
Overview effect	3.92	2.31	4.69	4.08	2.85	4.85	0.20	−1.00	0.79	−5.336**
Drawing size	5.40	0.20	9.30	5.30	0.40	8.80	0.00	−13.33	0.91	−.262
Correct answer	8	0	13	10	4	14	0.14	−1.25	0.70	−4.250**
Single-drawings	27	4	54	26	4	52	0.04	−3.50	0.76	−.721
Paired-drawings	27	3	58	34	3	58	0.21	−18.33	0.95	−.737

*p < .05, **p < .001

#### Relationship overview effect, awe, and learning gains

To assess the relationship between the overview effect, awe (drawing gains and annotated awe scores by raters), and learning gains, Spearman correlations were conducted. We predicted a positive correlation between the overview effect and annotated awe scores, and a negative correlation between drawing gains and annotated awe scores. Contrary to what we predicted, no significant correlations were found neither between the overview effect and drawing gains, *r*(98) = .178, *p* = .078, the overview effect and annotated awe scores for single drawings, *r*(98) = −.088, *p* = .388, nor for annotated awe scores for paired drawings, *r*(98) = .042, *p* = .682. Drawing gains did not significantly correlate with annotated awe scores for single drawings either, *r*(98) = .039, *p* = .705, nor with annotated awe scores for paired drawings, *r*(98) = .116, *p* = .253.

A positive correlation between learning, and annotated awe scores, and learning and the overview effect was expected. While learning gains did not significantly correlate with drawing size gains, *r*(98) = −.090, *p* = .377, they significantly correlated with annotated awe scores for single-drawings, *r*(98) = .344, *p* < .001, and annotated awe scores for paired drawings, *r*(98) = .378, *p* < .001. See Table 2 for an overview of the correlations. This indicates that participants with higher learning gains also had a higher annotated score for awe on their drawings.

**Table 2:** Overview of Spearman correlations

	1	2	3	4
1) Learning gains				
2) Overview effect	−.049			
3) Drawing size gains	−.090	.178		
4) Single-drawing gains	.344**	−.084	.039	
5) Paired-drawings gains	.378**	−.140	.116	.813**

*p < .05, **p < .001

#### Rating study on drawings

To assess which elements in the drawings were associated with awe, raters’ descriptions of what aspects in a drawing they paid attention to while scoring the drawings was analyzed. Mentioned most frequently in their reports were the words *details* (36% of raters), and *other elements* (17% of raters) such as *space* (28% of raters), *aliens* (16% of raters), *planets* (17% of raters), and *astronaut* (12% of raters), and facial expressions or emotions (27.5% of raters). When visually comparing top scoring and bottom scoring drawings, the ones with fewer details (e.g., where a drawn character was represented as a stick figure) received a lower annotated score for awe than drawings with a lot of elements and details in it. This suggested that raters considered *details* and different types of *other elements* to depict more awe.

#### Details

To further investigate the *details* as pointed out by the raters, drawings were scored by the authors on having other elements than just the drawn astronaut (1), or not (0). While the number of participants showing details increased from 36 in the pretest to 39 in the posttest, an exact McNemar’s test determined that there was no significant difference for the VR intervention, *p = .*710. Upon further analysis, the difference between two drawings (post–pre) on *details* correlated significantly with drawing size gains in the expected direction, *r*(99) = −0.25, *p* = .011, as well as with the annotated awe score for single drawings, *r*(99) = .42, *p* < .001, and the annotated awe score for paired drawings, *r*(99) = 0.42, *p* < .001. This indicates that when human figure drawings are smaller, there are more details and other elements on the drawings and are more often rated as portraying awe.

#### Complexity

Drawings were next scored by the authors on the nature of the character being drawn—that is, whether they were stick figures (1) or not (0), reflecting simplicity. The number of participants drawing stick figures decreased from 22 in the pretest to 13 in the posttest, which was a significant difference as shown by an exact McNemar’s test, *p = .*022. The difference between two drawings (post–pre) correlated significantly with the annotated awe score for paired drawings in the expected direction, *r*(99) = −.20, *p* = .044. This indicates that when a stick figure is drawn, it is likely that raters do not score it as portraying awe. The correlation with the annotated awe score for single drawings was in the expected direction as well but did not reach significance, *r*(99) = −0.14, *p* = .155.

### Structural equation model

**Fig. 2:**
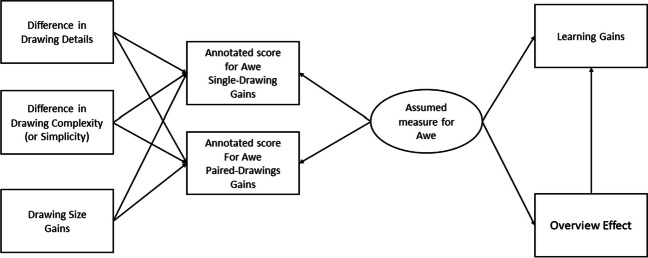
Hypothesized SEM

**Fig. 3:**
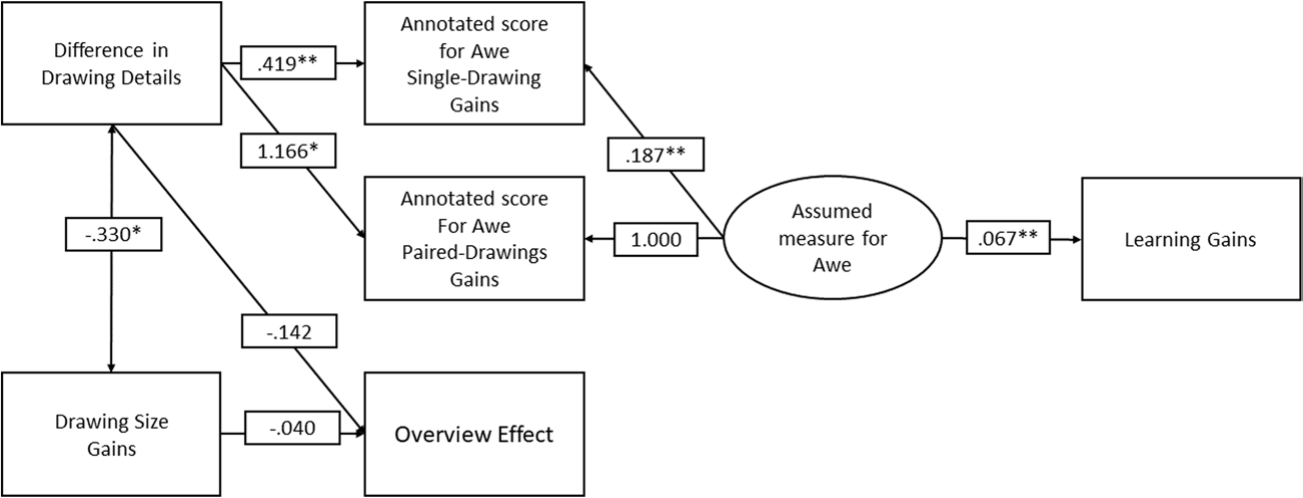
Final SEM, including estimates

To assess awe as a latent variable from drawing size, drawing details, drawing complexity, both of the rating studies, its effect on the overview effect, and learning gains, a structural equation model (SEM) was used. Since the data was not normally distributed, results were confirmed using bootstrapping. Results were calculated using maximum likelihood, with estimated means and intercepts. Bootstrapping was performed with 5,000 samples, with a 95% BC bias-corrected confidence level. In the hypothesized model, it was assumed that all drawing elements influenced the rater scores. The model did not reach an acceptable fit (*CFI* = .875, *TLI* = .738, *RMSEA* = .120), and can be seen in Fig. 2. Because of the relation between the overview effect, the drawings, and awe, the position of the overview effect was changed within the model to assess whether drawing elements influence it in another way. Subsequently, variables and connections that did not contribute to the model were removed, until the model had an acceptable fit (*CFI* = 1.000, *TLI* = 1.005, *RMSEA* = .000); the final model can be seen in Fig. 3.

The pre- to post- difference in drawing details showed a significant positive relation to the annotated scores for awe (single-drawing gains, *β* = .419, *p <* .001, paired-drawings gains, *β* = 1.166, *p =* .015). This variable negatively related to the overview effect (*β* = −.142, *p =* .070); however, this did not reach significance. Drawing size and drawing details were significantly correlated in the expected direction (*β* = −.330, *p =* .003), and drawing size had a negative relationship with the overview effect (*β* = −.040, *p =* .063); however, this did not reach significance. The combination of the annotated scores for awe seemed to explain the assumed measure for awe, the annotated awe scores (single-drawing gains, *β* = .187, *p <* .001, paired-drawing gains, *β* = 1.000). Finally, the assumed measure for awe significantly related to learning gains (*β* = .067, *p <* .001). All results were confirmed by bootstrapping. For a full overview of estimates and confidence intervals of the model, see Table 3.

**Table 3:** Overview SEM estimates model

	*β*	*SE*	Bootstrap 95% CI
Annotated score paired-dr. gains ← Assumed Awe	1.000		[1.000;1.000]
Learning gains ← Assumed Awe	.067**	.019	[.025; .117]**
Annotated score single-dr. gains ← Assumed Awe	.187**	.042	[.097; .282]**
Annotated score single-dr. gains ← Drawing details	.419**	.120	[.196; .672]**
Annotated score paired-dr. gains ← Drawing details	1.166*	.480	[.461; 2.031]**
Overview Effect ← Drawing details	−.142	.078	[−.307; .004]
Overview Effect ← Drawing size gains	−.040	.022	[−.068; .010]
Drawing details ←→ Drawing size gains	−.330*	.112	[−.763; −.068]**

*n* = 99, bootstrapping *N* = 5000, *p = .05, **p < .001

## Discussion

The purpose of this study was to gain an understanding of the relationship between self-representation and content in children’s drawings and the transformative experience of awe elicited by means of a virtual reality simulation of the overview effect. In line with Bai et al. (2017), we hypothesized a reduction in drawn self-size due to experiencing the overview effect. In addition to self-size, the drawings were assessed by independent raters on elements likely expressing the emotion of awe, to investigate the potential of drawings as an additional method next to questionnaires. We predicted that the simulated overview effect experience would result in knowledge acquisition, since awe is considered an epistemic emotion (van Limpt-Broers et al., 2020a, b) that triggers a need for accommodation of new information as well (Keltner & Haidt, 2003).

Congruent with previous studies using immersive simulations to induce such a transformative experience (Chirico et al., 2017; Gallagher et al., 2014; McPhetres & Shtulman, 2021; Quesnel & Riecke, 2017; van Limpt-Broers et al., 2020a, b), evidence was obtained that the overview effect was successfully induced, with a higher score on the posttest compared with the pretest. Furthermore, the statistical analysis of learning gains from pre- to posttest showed that the VR simulation resulted in the expected knowledge acquisition (Anderson et al., 2020; Cuzzolino, 2019; McPhetres, 2019; van Limpt-Broers et al., 2020b).

The results from van Limpt-Broers et al. (2020b) showed dispositional awe to be related to the overview effect, which in turn yielded learning. However, the current study did not reveal a relation between the overview effect and learning gains. The link of awe between these two variables appears to be missing. Raters annotated drawings for the presence or absence of awe, but instead of the expected correlation between this assumed measure for awe and the overview effect, the annotated drawing scores linked to learning gains instead. This was confirmed using the SEM. Both this information and the fact that more details in drawings were associated with how raters think awe is portrayed, suggests that it was not the emotion of awe that was being measured by raters but something else, such as creativity or cognitive ability. Even though this might suggest that drawings cannot be used for these constructs, other human figure drawing methods were reported to evaluate the assessment of cognitive ability or intelligence (Imuta et al., 2013; Willcock et al., 2011).

The present study did not reveal evidence for the relation between awe and the small-self as measured with drawn self-figures. This in itself is an important finding, considering that self-size might not be representational for awe for this age group, contrary to what was predicted based on Bai et al. (2017). But there may also be other explanations, such as children being influenced by the size of the elements within the drawing prompt that they might have attempted to proportionally match. Note that Bai et al. (2017) did not use pre and post measurements as employed in the current study, and perhaps this may have affected our results (as it may have affected theirs). Another possible explanation of these findings might be that while self-size representation could be reduced upon viewing an awe-inspiring stimulus (Bai et al., 2017), the drawing of self-figures may be culturally dependent (La Voy et al., 2001; Yap et al., 2022) or perhaps age-dependent and not applicable to children between 10 and 12 years of age.

The lack of difference in drawn self-size resulted in an assessment by independent raters who annotated children’s drawings for portraying awe or not, which also did not indicate any significant changes between the two moments of measurement. Even though we found no evidence for small-self in children’s drawings as a measure for awe, we did find additional information regarding what people look for in drawings when assessing transformative experiences. Independent raters used *details*, complexity, and *facial expressions* as potential indicators for awe. Drawing clear facial expressions may be culturally dependent as well, however, and it is not possible to tell whether the presence of facial expression is a result of cultural values or felt emotions with this mixed participant sample of an International School (La Voy et al., 2001).

While the number of details in a drawing correlated with annotated awe scores by raters, it *negatively* correlated with self-size, meaning that when the number of details on a drawing increased from pre- to posttest, self-size decreased. Since awe is related to smaller size it could indicate that the attention was moving away from the self, and towards the environment, suggesting a unitive experience (Nour et al., 2016). However, more details on a drawing might also simply indicate that more space is taken up in the drawing by other elements and leave less space for the human figure. There was no significant difference in details from pre- to postdrawings, which means that this might not be a good indicator for an awe experience.

Drawings in the posttest included more complexity (or less simplicity) than in the pretest, thereby providing a clue to visual representations of experienced awe in children’s drawings. Complexity in stimuli is known to evoke awe, which could be a reason why this is reflected in drawings associated with awe (Luke, 2021). Another reason why more complexity could be present in awe-associated drawings is that this emotion may lead to more creativity (Chirico, Glaveanu, et al., 2018b; Zhang et al., 2021).

The use of raters annotating drawings as a proxy for extracting content information and meaning is not uncommon (Cox et al., 2001; Groth-Marnat & Roberts, 1998; Jurovatý et al., 2022). However, in the current research it was not possible to retroactively ask the children participants for the true meaning behind their drawings and drawing elements. While rater interpretation is insightful regarding transformative experiences, it was not matched to participants’ intent, which is advised for future studies.

The reliability of data could have been further influenced by participant age, perhaps explaining the missing data points within the questionnaires as questions are easily skipped, as well as student mood and motivation (Borgers et al., 2000). We tried to take these factors into account when designing the experiment by adjusting the number of questionnaires to convene with assumed participants’ attention span, and ability (Bell, 2007; Borgers et al., 2000; Van Kesteren et al., 2003). Future studies can extend the current findings to various age groups and perhaps link this research to a measure and comparison between awe and cognitive ability (Imuta et al., 2013; Willcock et al., 2011).

Despite these limitations, the present study enhances our understanding of the relationship between transformative experiences and self-representation in children’s drawings. Where the focus of pictorial measures on awe in previous research was mainly on self-size (Bai et al., 2017; Colantonio & Bonawitz, 2018; Van Elk et al., 2016; Sawada & Nomura, 2020; Sturm et al., 2020), our results suggest that self-size might not be a good indicator for this emotion in younger participants. Other elements, such as drawing complexity, should be considered when measuring transformative experiences.

## Figures and Tables

**Fig. 4:**
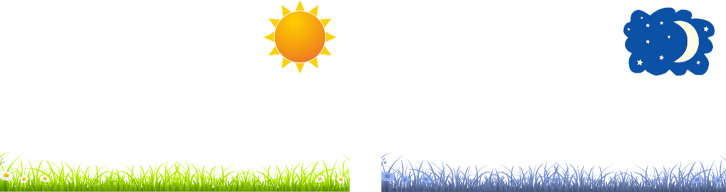
Drawing field Version 1 and Version 2

## Data Availability

The datasets generated during and analyzed during the current study are publicly available under license CC-BY-NC-ND-4.0, through the DataverseNL repository (10.34894/HV9U9Z).

## Appendix 1

**Table 4:** Overview effect—Positive effects of being in space questionnaire

1	Original	I gained a stronger appreciation of the Earth’s beauty.
	*Version 1*	*Do you think the Earth is pretty?*
	*Version 2*	*Do you think the Earth is beautiful?*
2	Original	I learned to appreciate the fragility of Earth.
	*Version 1*	*How easily do you think the Earth can be destroyed?*
	*Version 2*	*How simply do you think the Earth can be damaged?*
3	Original	I realized how much I treasure Earth.
	*Version 1*	*Do you value Earth?*
	*Version 2*	*Do you treasure Earth?*
4	Original	I increased my involvement in environmental causes.
	*Version 1*	*Do you take good care of the Earth (such as cleaning up garbage, or turning off the lights when you leave your room?*
	*Version 2*	*Do you take good care of the environment (such as throwing things in the trash, or turning off the tap while brushing your teeth)?*
5	Original	I gained a stronger sense of wonder about the universe.
	*Version 1*	*Are you amazed by space, the Earth, and the planets?*
	*Version 2*	*Do you have a sense of wonder about the Earth, the planets, and space?*
6	Original	I became more excited about space exploration.
	*Version 1*	*Do you enjoy learning about the unknown parts of space?*
	*Version 2*	*Are you excited about new things being discovered in the universe?*
7	Original	I gained a new appreciation for the boundlessness of the Cosmos.
	*Version 1*	*Do you find it special that the universe has no borders?*
	*Version 2*	*Do you find it extraordinary that space is infinite?*
8	Original	I became interested in the possibility of life on other planets.
	*Version 1*	*Do you want to learn about the possibility of life on other planets?*
	*Version 2*	*Do you want to know more about the possibility of alien life?*
9	Original	My relationship with my family grew stronger
	*Version 1*	*Do you have a good relationship with your family?*
	*Version 2*	*Do you get along well with your family members?*
10	Original	I gained a stronger appreciation for the unity of humankind.
	*Version 1*	*Do you think it is important that all people on Earth get along?*
	*Version 2*	*Do you think it is important that all people on Earth can work well together?*
11	Original	I was inspired to express my creativity.
	*Version 1*	*Do you show how creative you are?*
	*Version 2*	*Do you show how artistic you are?*
12	Original	I increased my involvement in political activities.
	*Version 1*	*Do you want to know more about what the government does?*
	*Version 2*	*Do you want to learn about what political parties do?*
13	Original	I began to think that differences in political ideology are arbitrary.
	*Version 1*	*Do you take it for granted that people in politics have different views?*
	*Version 2*	*Do you think it makes sense that people in the government have different opinions?*

## Appendix 2 Awe—Draw yourself as an astronaut

Instructions:

Draw yourself in the image below. You can draw anything you want, and the picture can be anything, as long as you are in it.

## Footnotes

1 Other mechanisms that lead to similar feelings of the small-self are psychedelics (Nour et al., 2016), mental disfunction (Lebedev et al., 2015), spiritual or near-death experiences (Martial et al., 2021; Taves, 2020; Taylor, 2018), and meditation (Stoliker et al., 2021), all of which lead to detachment from the self, an outward focus, and a quiet mind (Taylor, 2018) or thoughtless emptiness (Hinterberger et al., 2014).

2 The full simulation, or a recording of it, cannot be shared because of copyrighted material owned by SpaceBuzz. Parts of the simulation can be viewed on YouTube through https://www.spacebuzz.earth/

3 To clarify, Cronbach’s alpha was calculated per questionnaire version. The versions were administered separately as a pre- and postmeasures, and synonymous questions were not repeated within one session. If questionnaires would have been merged together, a higher validity would have been achieved because of a larger number of similar questions, resulting in an alpha of .83.

## Declarations

### Preregistration statement

The experiment was not preregistered.
